# Comparison of the regeneration of cartilage and the clinical outcomes after the open wedge high tibial osteotomy with or without microfracture: a retrospective case control study

**DOI:** 10.1186/s12891-019-2607-z

**Published:** 2019-06-01

**Authors:** O-Sung Lee, Seung Hoon Lee, Su Jung Mok, Yong Seuk Lee

**Affiliations:** 1 Department of Orthopaedic Surgery, Mediplex Sejong Hospital, Incheon, South Korea; 2 Department of Orthopaedic Surgery, Incheon Metropolitan City Medical Center, Incheon, South Korea; 3 Department of Orthopaedic Surgery, Seoul National University College of Medicine, Bundang Hospital, Seoul, South Korea

**Keywords:** Knee, Open wedge high tibial osteotomy, Microfracture, Cartilage, Regeneration

## Abstract

**Background:**

It is unclear whether postoperative outcomes are associated with the cartilage regeneration after open wedge high tibial osteotomy (OWHTO) combined with microfracture. The purpose of this study was to evaluate the regeneration of the articular cartilage, radiologic, and clinical outcomes after OWHTO with and without microfracture.

**Methods:**

Eighty-seven patients who underwent OWHTO from 2014 to 2015 were retrospectively included in this study. Fifty-seven OWHTOs with microfracture on medial femoral condyle (MFC) (group 1) and 30 OWHTOs without microfracture (group 2) were compared at a mean 2-year follow-up. The regeneration of the articular cartilage was evaluated using International Cartilage Repair Society (ICRS) grade on the second-look arthroscopy and the magnetic resonance observation of cartilage repair tissue (MOCART) score on magnetic resonance imaging (MRI). The weight-bearing line (WBL) ratio, hip-knee-ankle (HKA) angle, joint line convergence angle (JLCA) and Ahlbäck grade were evaluated. The clinical outcomes were evaluated using the Western Ontario and McMaster University (WOMAC) scores and the Knee Society (KS).

**Results:**

The articular cartilage in the MFC were regenerated in 67.8% of group 1 (43/57) and 58.6% of group 2 (16/30), respectively (*p* = 0.014). However, change of the ICRS grades of the medial tibial plateau, lateral and patellofemoral compartments showed no statistical difference between the groups. Total MOCART score in group 1 was superior to that in the group 2 at postoperative 2 years (41.8 ± 18.6 vs. 31.8 ± 19.8, *p* = 0.023). Regarding MOCART score, microfracture was only effective in the defect filling and integration to the border zone of the MFC (*p* < 0.001 and *p* = 0.035, respectively). Other radiologic and clinical outcomes showed no statistical differences between the groups.

**Conclusion:**

Microfracture of the MFC during OWHTO only helped the filling of the degenerative cartilage defect and the integration of the cartilage with adjacent cartilage. However, the clinical and radiologic outcome could not be improved by mircrofracture in the OWHTO.

## Background

Open wedge (OW) high tibial osteotomy (HTO) is an effective surgical treatment for patients with medial compartmental osteoarthritis combined with varus alignment [1–4]. The correction of the varus alignment by OW HTO provides change of the load distribution in the knee joint and improved functionality in patients with medial compartment osteoarthritic knees [5, 6]. Additionally, reduced load on the medial compartment due to lateral shift of the axial load has been shown to lead to biological regeneration of the articular cartilage after OW HTO [7–9]. However, lateral and patellofemoral compartments could be affected and changes of the cartilage status of these compartments are also questionable.

Although excellent short- to mid-term results after OW HTO have been reported in terms of clinical and radiologic results, clinical and radiological deterioration has been observed over long-term follow-up [2, 10, 11]. Therefore, several studies have suggested that OW HTO combined with cartilage repair techniques such as microfracture, subchondral drilling, abrasion arthroplasty, and autologous chondrocyte implantation might enable more effective cartilage regeneration and improved long-term outcomes [12–15]. Among them, microfracture has been one of the most commonly used procedures for cartilage repair procedures [14, 16–18]. Mocrofracture creates multiple holes in the subchondral bone in order to stimulate bone marrow. The cartilage defects are filled with precursor cells, resulting in a new cartilage and regenerative tissue [14]. However, it is unclear whether postoperative clinical outcomes are associated with the quality of cartilage regeneration after OW HTO combined with microfracture. Furthermore, little is known about the factors that influence clinical outcomes after OW HTO.

The purpose of this study was to compare the regeneration of the articular cartilage, radiologic, and clinical outcomes after OW HTO with and without microfracture. Our hypotheses were that the articular cartilage in the medial compartment after OW HTO with microfracture would be regenerated better than that without microfracture regardless of the radiologic and clinical outcomes of both groups [19].

## Methods

### Patients

This retrospective case-case control study was carried out between between March 2014 and May 2015. Patients received an arthroscopy at the time of OW HTO. We performed microfracture only when the medial femoral condyle (MFC) hadd a full-thickness articular cartilage lesion with a ballotable and unstable flap of cartilage or a partial thickness articular cartilage lesion of which the cartilage was simply scraped off down to the bone when probed. Microfracture was not performed if the patient had an overly large full-thickness lesion with an evenly distributed and stable calcified layer or if the surrounding cartilage was stable. All patients underwent removal of the locking plate with second-look arthroscopy at around 2 years after OW HTO. This study included only patients who completed radiologic and clinical assessment at the time of locking plate removal.

The inclusion criteria for OW HTO were: (1) primary osteoarthritis (not inflammatory arthritis), (2) radiographs showing medial-compartment knee osteoarthritis of Ahlbäck grades 1 to 3, [20] (3) concurrent varus alignment of operated limb, and (4) the patients who failed to respond to non-surgical treatments such as weight loss, physical therapy, activity modification, and drugs.

**Fig. 1:**
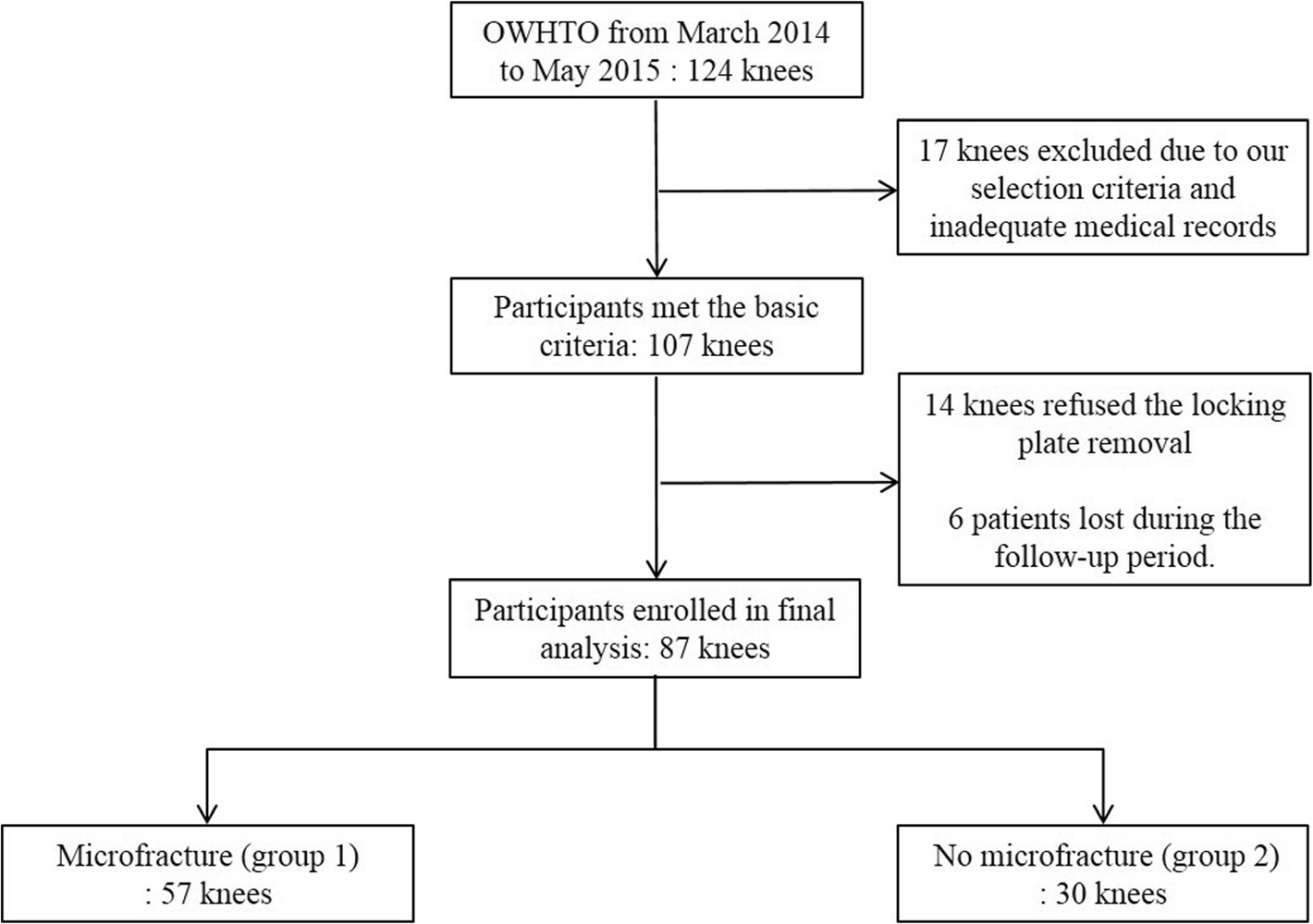
Study cohort and design (OW HTO, open wedge high tibial osteotomy)

From March 2014 to May 2015, a total of 124 knees underwent OW HTO. Among them, 17 patients excluded from this study due to our exclusion criteria and inadequate medical records: (1) secondary arthritis such as post-traumatic arthritis, (2) OW HTO associated with additional surgery such as anterior cruciate ligament reconstruction, (3) concurrent bilateral OW HTO, (4) revision OW HTO, (5) double osteotomy including distal femoral osteotomy, and (6) the patient whose standing radiograph in full knee extension could not be obtained because of the limitation of extension. Additionally, we couldn’t conduct second-look arthroscopy in 14 patients and 6 patients were lost during follow-up period. Final drop-out rate was 29.8% and 87 patients were successfully included in this study. Fifty-seven OW HTOs with microfracture on the MFC (group1) and 30 OW HTOs without microfracture (group2) were compared with a mean follow-up of 2 years (mean 2.0 ± 1.6) (Fig. 1). This retrospective study obtained the approval of the institutional review board of our hospital.

### Surgical technique and rehabilitation

All surgical procedures were performed by a single surgeon. The target mechanical axis was the weight-bearing line passing through 62.5% of the width of the tibial plateau, corresponding to a postoperative mechanical valgus of 2° to 4°. All patients received an arthroscopy at the time of OW HTO. Arthroscopic procedures included joint debridement, meniscectomy of a medial meniscus tear, removal of loose bodies and microfracture. We trimmed any loose flaps of articular cartilage and debrided the subchondral bone. The holes were made with an arthroscopic awl perpendicularly through the exposed subchondral bone, at which point bleeding from the holes was observed. After the microfracture, a longitudinal incision about 5-cm long was made at the anteromedial aspect of the proximal tibia. After release of the superior border of the pes anserinus and the anterior border of the medial collateral ligament, horizontal osteotomy was performed, and an additional biplanar anterior osteotomy was performed. The gradual distraction was performed at the most posterior portion of the osteotomy gap until the target limb alignment was obtained [21]. The osteotomized bone was fixed with a long locking plate (DWLP, TDM, Seongnam, Korea). The osteotomy gap was then filled with a bone graft substitute and no intra-articular drain was inserted.

In terms of postoperative rehabilitation, tolerable weight-bearing with crutches was encouraged for the patients with no microfracture, and they were allowed full weight-bearing, immediately postoperatively. However, the patients with microfracture treatment were allowed partial weight-bearing at postoperative 2 weeks and full weight-bearing at postoperative 4 weeks. All patients performed a passive exercise for range of motion of the operated knee at 2 days after surgery until a maximum flexion angle of 130° or more was achieved. Squatting with weight was restricted for 3 months, and patients were instructed to be careful when getting up from the sitting position.

### Evaluation of cartilage regeneration

The articular cartilage of the MFC, medial tibial plateau (MTP), lateral femoral condyle (LFC), lateral tibial plateau (LTP), patella, and trochlea was evaluated according to the International Cartilage Repair Society (ICRS) grading system by an arthroscopy at the time of OW HTO. The regeneration of the articular cartilage was also evaluated according to the ICRS grading system by second-look arthroscopy at the time of plate removal [22]. The ICRS grading system classified a macroscopically normal cartilage without notable defects as ICRS 0, a cartilage with a fibrillated, slightly softening surface or an superficial fissures as ICRS 1, a defect < 50% of the cartilage thickness as ICRS 2, a defect > 50% of the cartilage thickness as ICRS 3, and a full-thickness osteochondral injury as ICRS 4.

In addition, all patients underwent postoperative magnetic resonance imaging (MRI) to assess the quality of cartilage regeneration on the day before plate removal. Routine MRI protocol included fast-spin echo (dual T2-FSE) and fat-suppressed gradient echo (3D-GE-FS) sequences with 3-Tmagnetic resonance system (Ingenia, Philips Healthcare, Best, Netherlands). Morphological evaluation of the postoperative articular cartilage status on the MFC was performed according to a modified magnetic resonance observation of cartilage repair tissue (MOCART) scoring system by 2 independent investigators in a blinded manner [23]. A MOCART score of 100 indicates the best articular cartilage status and 0 indicates the worst status [24].

### Evaluation of radiologic and clinical outcomes

Clinical and radiographic evaluations were performed preoperatively and at the time of second-look arthroscopy. The weight-bearing line (WBL) ratio, hip-knee-ankle (HKA) angle, joint line convergence angle (JLCA) and Ahlbäck grade were measured preoperatively and at the time of second-look arthroscopy on full-length standing anteroposterior radiographs. The INFINITT version 5.0.9.2 (INFINITT, Seoul, South Korea) was used for the radiographic measurements. The HKA angle was measured as the angle between the line from the center of the femoral head to the center of the knee joint and the line from the center of the knee joint to the center of the ankle joint. A WBL was defined as the line drawn from the center of the femoral head to the center of the superior surface of the talus. The denominator of WBL ratio was the width of the tibial plateau, and the numerator of WBL ratio was the medial tibial intersection of the WBL in the knee joint (the medial tibial edge at 0% and the lateral tibial edge at 100%). The JLCA was defined as the angle between the line connecting the articular surfaces of the distal femur and the proximal tibia. Additionally, the Ahlbäck classification was used for the evaluation of the radiologic severity of osteoarthritis [20]. Flexion contracture and active maximal flexion were measured in the supine position using a goniometer. The clinical status of each knee was rated using the Western Ontario and McMaster University (WOMAC) scores and the Knee Society (KS) knee and functional scores.

### Statistical analysis

Statistical analyses were performed with SPSS version 22.0 statistical package (IBM Corp., Armonk, NY, USA). Data was described based on means and standard deviation (SD) for continuous values. The differences in continuous variables were analyzed with Student’s t-test or the Mann–Whitney test according to the appropriate normality tests. The differences of other categorical variables were analyzed with Pearson’s chi-square test or Fisher exact test or linear by linear association. Univariate and multivariate regression analysis were performed to identify factors affecting the clinical results represented by KS knee and functional scores and WOMAC scores. All statistical significances were set at *p* < 0.05.

We determined a total sample size using a post hoc power analysis based on a previous study similar to this study [25]. A 10-point difference on the Knee Society scores was considered significant. An error and power were set to 5 and 80%, respectively using computer software (G*Power 3.1.0). The required sample size required was 33 knees per group, considering a possible dropout rate of 30%.

## Results

Among a total of 87 patients included in this study, 57 patients were included in group 1 (with microfracture) and 30 OWHTOs were included in group 2 (without microfracture), with a mean follow-up of two years. Both groups showed no statistical differences in terms of preoperative demographics. Additionally, no statistical differences between groups were observed with respect to WBL ratio, HKA angle, JLCA, Ahlbäck grade of osteoarthritis, range of motion, WOMAC scores and Knee scores (Table 1).

**Table 1:** Comparison of preoperative demographics and measurements

		Microfracture (*n* = 57)	No microfracture (*n* = 30)	*p*-value
Patients number		57	30	
Age (years)		57.0 ± 5.4	57.0 ± 6.5	0.989*
Sex (male/female)		37/20	7/23	0.333§
Side (Left: Right)		22/35	15/15	0.365§
Height (cm)		159.1 ± 8.1	156.8 ± 8.9	0.229*
Weight (kg)		67.4 ± 12.6	62.2 ± 10.2	0.403*
Body mass index (kg/m^2^)		26.5 ± 3.6	26.4 ± 3.3	0.950*
Onset of symptom (months)		34.7 ± 14.3	36.1 ± 25.3	0.897*
Follow up (years)		2.0 ± 0.2	1.9 ± 0.1	0.300*
Weight bearing line ratio (%)		19.1 ± 11.7	22.0 ± 12.3	0.153*
Hip-knee-ankle angle (°)		Varus 7.0 ± 3.1	Varus 6.2 ± 2.7	0.241*
Joint line convergence angle (°)		3.1 ± 1.7	2.8 ± 1.4	0.545*
Ahlbäck grade (grade 0/1/2/3/4)		0/42/13/2/0	0/26/4/0/0	0.162†
Flexion contracture (°)		2.5 ± 1.4	1.8 ± 2.8	0.393*
Active full flexion (°)		132.1 ± 7.2	134.0 ± 4.8	0.201*
WOMAC scores	Total	39.8 ± 13.2	39.5 ± 7.8	0.914*
	Pain	9.5 ± 3.5	8.4 ± 2.6	0.278*
	Stiffness	4.3 ± 2.1	4.2 ± 2.0	0.804*
	Function	26.0 ± 9.7	26.9 ± 8.7	0.674*
Knee scores	Knee scores	53.7 ± 17.0	52.7 ± 14.3	0.791*
	Function scores	59.5 ± 15.5	59.8 ± 9.0	0.925*

The values are presented as mean ± standard deviation. *Derived using the Student’s t-test. § Derived using the Pearson chi-square test. †Derived using the linear by linear association WOMAC; Western Ontario and McMaster Universities

The preoperative and postoperative assessments of articular cartilage according to the arthroscopic examinations are summarized in Table 2. The improvement of ICRS grade of MFC was observed in 75.4% (43/57) among the patients of group 1, whereas 53.3% (16/30) among the patients of group 2 showed the improvement of ICRS grade of MFC (*p* = 0.014). The improvement of MTP was observed in 61.4% (35/57) of group 1 and 53.3% (15/30) of group 2 with no statistical difference (*p* = 0.742). Otherwise, the articular cartilages of the LFC deteriorated in 38.6% (22/57) of group 1 and 26.9% (8/30) of group 2, respectively (*p* = 0.674), and that of the LTP deteriorated in 36.8% (21/57) of group 1 and 40.0% (12/30) of group 2, respectively (*p* = 0.936). Additionally, the articular cartilages of the patella and trochlea also showed similar deterioration (group 1 vs. group 2; 47.4% vs. 40.0%, *p* = 0.480; 45.6% vs. 63.3%, *p* = 0.496, respectively).

**Table 2:** Change in International Cartilage Research Society grade

ICRS grade on second look arthroscopy	Microfracture (*n* = 57)		No microfracture (*n* = 30)		*p*-value
	Preoperative	Postoperative	Preoperative	Postoperative	
MFC (Grade 0/1/2/3/4)	0/3/13/21/20	3/8/9/15/8/14	0/2/7/15/6	0/7/15/6/2/0	
Improvement/stationary/worsening	43 (75.4%) / 13 (22.8%) / 1 (1.8%)		16 (53.3%) / 10 (33.3%) / 4 (13.3%)		0.014*
MTP (Grade 0/1/2/3/4)	0/0/18/6/33	1/11/22/13/9/1	0/5/12/10/3	1/11/10/5/3/0	
Improvement/stationary/worsening	35 (61.4%) / 15 (26.3%) / 7 (12.3%)		15 (50.0%) / 13 (43.3%) / 2 (6.7%)		0.742*
LFC (Grade 0/1/2/3/4)	21/32/4/0/0	7/38/12/0/0	17/10/3/0/0	9/18/3/0/0	
Improvement/stationary/worsening	3 (5.3%) / 32 (56.1%) / 22 (38.6%)		0 (0.0%) / 22 (73.3%) / 8 (26.7%)		0.674*
LTP (Grade 0/1/2/3/4)	8/42/7/0/0	1/36/18/2/0	8/21/1/0/0	6/13/11/0/0	
Improvement/stationary/worsening	3 (5.3%) / 33 (57.9%) / 21 (36.8%)		2 (6.7%) / 16 (53.3%) / 12 (40.0%)		0.936*
Patella (Grade 0/1/2/3/4)	15/29/9/2/2	1/29/22/4/1	9/16/4/1/0	4/14/11/1/0	
Improvement/stationary/worsening	4 (7.0%) / 26 (45.6%) / 27 (47.4%)		3 (10.0%) / 15 (50.0%) / 12 (40.0%)		0.480*
Trochlea (Grade 0/1/2/3/4)	7/26/18/5/1	2/15/29/9/2	9/12/6/3/0	2/7/16/5/0	
Improvement/stationary/worsening	4 (7.0%) / 27 (47.4%) / 26 (45.6%)		4 (13.3%) / 7 (23.3%) / 19 (63.3%)		0.496*

The values are presented as number with the percent in parentheses. *Derived using the linear by linear association. The statistical significance was set at *p* < 0.05 derived using the linear by linear association. *MFC* medial femoral condyle, *MTP* medial tibial plateau, *LFC* lateral femoral condyle, *LTP* lateral tibial plateau

The mean MOCART score of the MFC mean at postoperative 2 years was superior in group 1 compared to that in the group 2 (41.8 ± 18.6 vs. 31.8 ± 19.8, *p* = 0.023). The number of knees showing complete filling, hypertrophy, > 50% of adjacent cartilage, < 50% of adjacent cartilage, subchondral bone exposure were 14, 16, 9, 15, 3 in group 1 and 2, 1, 5, 6, 16 in group 2, respectively (*p* < 0.001). In terms of the integration of repaired cartilage to the border zone, the number of knees showing complete integration, visible demarcating border, defect < 50% of the length of repair tissue, defect > 50% of the length of repair tissue were 3, 29, 16, 9 in group 1 and 3, 6, 9, 12 in group 2, respectively (*p* = 0.035) (Table 3). Although the microfracture of the MFC appeared to be helpful for cartilage healing according to the MOCART score, it was helpful only for the degree of defect repair and the integration to the border zone excluding other variables.

**Table 3:** Comparison of MOCART scores of medial femoral condyle between the microfracture group and no-microfracture group using MRI

Variable			Microfracture (*n* = 57)	No microfracture (*n* = 30)	*p*-value
Total score			41.8 ± 18.6	31.8 ± 19.8	0.023*
1. Degree of defect repair and filling of the defect	Complete		14	2	< 0.001†
	Hypertrophy		16	1	
	Incomplete				
	> 50% of adjacent cartilage		9	5	
	< 50% of adjacent cartilage		15	6	
	Subchondral bone exposed		3	16	
2. Integration to the border zone	Complete		3	3	0.035†
	Incomplete				
	Demarcating border visible		29	6	
	Defect < 50% of the length of repair tissue		16	9	
	Defect > 50% of the length of repair tissue		9	12	
3. Surface of the repair tissue	Surface intact		12	4	0.115†
	Surface damaged < 50% of repair tissue depth		29	12	
	Surface damaged > 50% of repair tissue depth		16	14	
4. Structure of the repair tissue	Homogeneous		7	4	0.998§
	Inhomogeneous or cleft formation		50	26	
5. Signal intensity of the repair tissue	Dual T2-FSE	Isointense	3	0	0.311†
		Moderately hypointense	17	8	
		Markedly hypointense	37	22	
	3D-GE-FS	Isointense	6	2	0.396†
		Moderately hypointense	21	9	
		Markedly hypointense	30	19	
6. Subchondral lamina	Intact		32	23	0.066§
	Not intact		25	7	
7. Subchondral bone	Intact		40	28	0.061§
	Edema		17	2	
8. Adhesions	No		49	28	0.483§
	Yes		8	2	
9. Effusion	No		48	22	0.261§
	Yes		9	8	

The values are presented as number except for the total score indicated. *Derived using the Student’s t-test. †Derived using the linear by linear association. §Derived with Pearson chi-square test. The statistical significance was set at *p* < 0.05. *MOCART* magnetic resonance observation of cartilage repair tissue

There were no statistically significant differences of the WBL ratio, HKA and JLCA between groups. Moreover, the severity of osteoarthritis indicated by the Ahlbäck grading system showed no statistical difference between groups (*p* = 0.699). All clinical scores showed no statistical difference between groups at a mean 2-year follow-up (Table 4).

**Table 4:** Comparison of postoperative results between microfracture group and no microfracture group

		Microfracture (*n* = 57)	No microfracture (*n* = 30)	*p* value
Weight bearing line ratio (%)		58.6 ± 7.2	57.5 ± 6.2	0.060*
Hip-knee-ankle angle (°)		Valgus 2.3 ± 1.4	Valgus 1.9 ± 1.6	0.270*
Joint line convergence angle (°)		1.9 ± 1.7	2.1 ± 2.1	0.705*
Ahlbäck grade (grade 0/1/2/3/4)		11/2/38/7/1	5/23/2/0/0	0.699†
Flexion contracture (°)		2.2 ± 1.2	1.8 ± 1.6	0.403*
Further flexion (°)		134.9 ± 7.2	136.0 ± 6.6	0.492*
WOMAC scores	Total	9.2 ± 6.1	10.2 ± 7.8	0.513*
	Pain	1.3 ± 1.2	1.7 ± 1.6	0.278*
	Stiffness	1.1 ± 1.4	1.3 ± 1.5	0.433*
	Function	6.7 ± 4.5	7.2 ± 5.9	0.678*
KS scores	KS knee scores	89.1 ± 10.7	88.3 ± 10.8	0.745*
	KS function scores	88.3 ± 10.8	86.1 ± 12.3	0.634*

The values are presented as mean ± standard deviation. *Derived using the Student’s t-test. †Derived using the linear by linear association. *WOMAC* Western Ontario and McMaster Universities

The results of univariate and multivariate regression analysis for evaluating factors affecting clinical scores after OW HTO are shown in Table 5. The univariate analysis showed that the postoperative values of HKA angle, WBL ratio, JLCA, Ahlbäck grade and postoperative cartilage healing of MFC and MTP were significantly related with the postoperative clinical scores at a mean 2-year follow-up. However, in mulivariate analysis, only postoperative WBL ratio was significantly associated with postoperative KS knee score and WOMAC pain score (*p* = 0.010 and *p* = 0.045, respectively).

**Table 5:** Regression analysis of clinical scores

Univariate regression analysis	KS knee	KS function	WOMAC pain	WOMAC stiffness	WOMAC function	WOMAC total
Age	0.809	0.539	0.549	0.962	0.585	0.639
BMI	0.476	0.204	0.207	0.776	0.277	0.188
Sex	0.398	0.597	0.075	0.259	0.877	0.972
Duration of symptom	0.906	0.599	0.07	0.455	0.751	0.629
Site of microfracture	0.636	0.514	0.118	0.349	0.788	0.765
Size of microfracture	0.244	0.729	0.568	0.300	0.629	0.804
Preoperative HKA angle	0.982	0.95	0.447	0.818	0.204	0.27
Postoperative HKA angle	**0.003**	**0.002**	**< 0.001**	0.52	0.053	**0.006**
Preoperative WBL ratio	0.873	0.903	0.183	0.828	0.093	0.106
Postoperative WBL ratio	**< 0.001**	**< 0.001**	**< 0.001**	0.396	0.089	**0.009**
Preoperative JLCA	0.069	0.057	0.677	0.703	0.926	0.919
Postoperative JLCA	**0.031**	**0.006**	**0.002**	0.73	0.122	0.077
Preoperative Ahlbäck grade	0.495	0.356	0.607	0.726	0.284	0.28
Postoperative Ahlbäck grade	**0.042**	0.073	0.089	0.964	0.526	0.382
Cartilage healing of MFC	**0.001**	**0.001**	**< 0.001**	0.443	0.296	0.098
Cartilage healing of MTP	**0.004**	**0.002**	**0.002**	0.326	0.485	0.894
Multivariate regression analysis	KS knee	KS function	WOMAC pain	WOMAC total		
Postoperative HKA angel	0.126	0.327	0.754	0.086		
Postoperative WBL ratio	**0.010**	0.06	**0.045**	0.892		
Postoperative JLCA	0.652	0.635	0.806			
Postoperative Ahlbäck grade	0.654					
Cartilage healing of MFC	0.055	0.184	0.089			
Cartilage healing of MTP	0.582	0.337	0.686			

*HKA* hip-knee-ankle, *WBL* weight bearing line, *JLCA* joint line convergence angle, *MFC* medial femoral condyle, *MTP* medial tibial plateau, *KS* Knee Society, *WOMAC* Western Ontario and McMaster Universities

## Discussion

The principal findings of this study were that microfracture of the MFC during OW HTO improved the regeneration of the articular cartilage of the MFC, whereas those in the lateral and patellofemoral compartments were found to have deteriorated at a mean 2-year follow-up. It was only effective with respect to the degree of defect repair (filling of the defect) and integration to the border zone of the MFC. Additionally, microfracture could not improve the radiologic and clinical outcomes after the OW HTO.

Several authors reported the regeneration of degenerated articular cartilage after HTO in the absence of any cartilage repair procedure [8, 9, 26, 27]. Fujisawa et al. [26] reported that the cartilage lesion was covered with fibrous and membranous tissue about 1.5 to 2 years after osteotomy if ideal correction was obtained. Koshino et al. [9] reported that 133 of 146 cases (91%) achieved partial or total coverage with fibrocartilage after closed wedge HTO. However, cartilage repair procedures such as microfracture, subchondral drilling, and abrasion arthroplasty in conjunction with HTO have reportedly contributed to more favorable clinical and histological outcomes [14, 18, 25]. Among them, microfracture has been commonly performed with OW HTO by many surgeons because of its safety and convenience. However, the effect of microfracture on clinical outcomes has been controversial [14, 16–18]. In terms of clinical outcomes, Sterett et al. [28] reported excellent survival rates of 91% survivorship at 7 years after microfracture with OW HTO. Pascale et al. [18] reported that patient satisfaction was increased among those who underwent HTO plus microfracture compared with those who underwent HTO alone at the 5-year follow-up. However, Matsunaga et al. [14] reported that microfracture combined with HTO did not lead to superior clinical outcomes compared to HTO alone at 1, 3, and 5 years postoperatively. Moreover, Ferruzzi et al. [16] reported that microfracture associated with HTO provided the worst Hospital for Special Surgery scores and WOMAC scores compared to both HTO alone and HTO with autologous chondrocyte implantation.

In terms of the macroscopic healing of the articular cartilage after OW HTO, second-look arthroscopies have been found to confirm the regeneration of degenerated articular cartilage in the medial compartment after HTO without any cartilage regeneration techniques [8, 9, 29]. Indeed, Jung et al. [8] reported that the regeneration of degenerated cartilage of the MFC was found in 75–92% of patients after OW HTO without any cartilage regeneration strategies. Kim et al. [29] also reported that lesions in the MFC and the MTP of 104 HTO knees were improved in 54 knees (51.9%) and 36 knees (34.6%) without additional cartilage regeneration techniques. Moreover, Matsunaga et al. [14] reported that the second-look arthroscopy revealed better regeneration of the distal femur cartilage in the patients with the abrasion arthroplasty with HTO than those with either HTO alone (*p* < 0.0005) or microfracture with HTO (*p* < 0.01), with no difference between the 2 approaches.

**Fig. 2:**
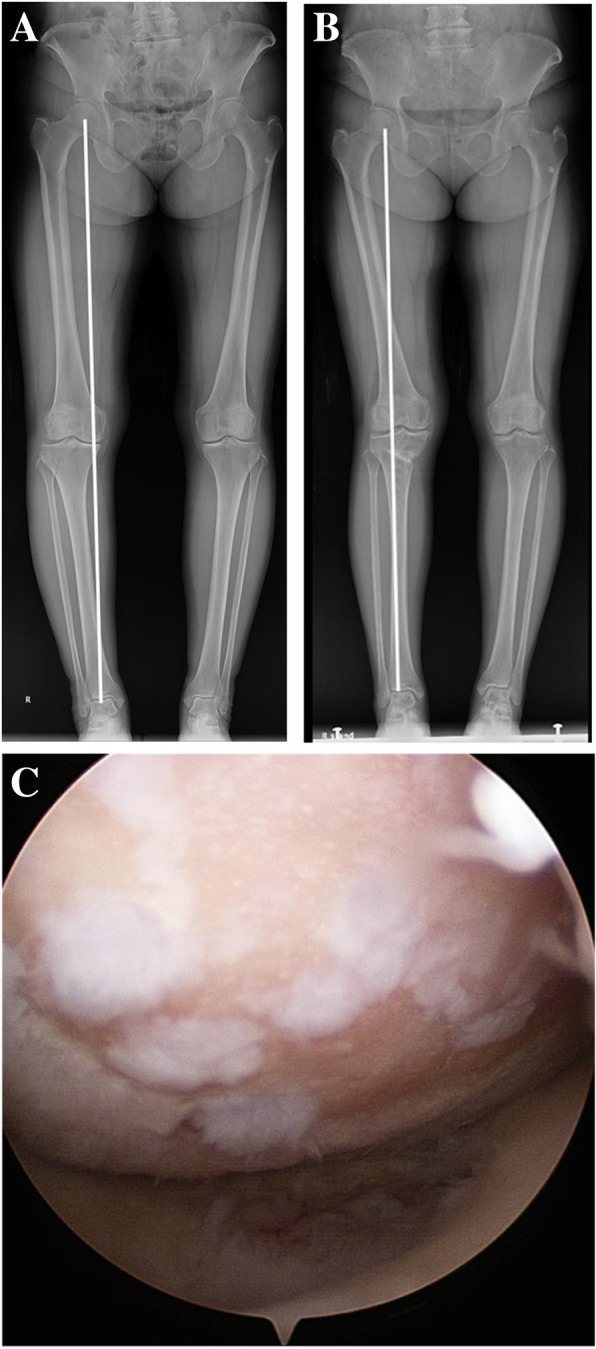
Images from a 59-year-old woman show the following: (**a**) Ahlbäck grade I osteoarthritis with varus alignment of the lower limb before open wedge high tibial osteotomy with microfracture of the medial femoral condyle and medial tibial plateau; (**b**) Postoperative alignment of the lower limb obtained a satisfactory WBL ratio (64.5%). However, there was no recovery of the joint space narrowing; and (**c**) second-look arthroscopy shows insufficient coverage of the articular cartilage at postoperative 25 months. However, the Knee Society knee score significantly improved from 55 points preoperatively to 95 points, and the Western Ontario and McMaster Universities pain score significantly improved from 13 points preoperatively to 1 point at the time of second-look arthroscopy

**Fig. 3:**
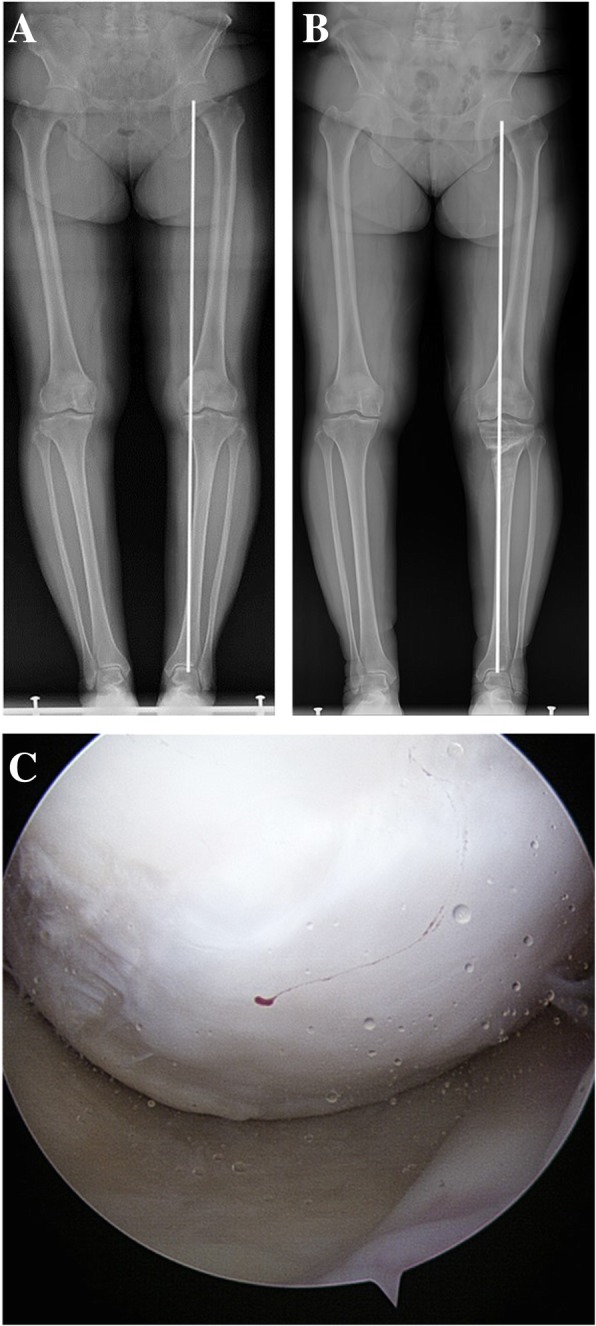
Images from a 55-year-old woman show the following: (**a**) Ahlbäck grade I osteoarthritis with varus alignment of the lower limb before open wedge high tibial osteotomy with microfracture of the medial femoral condyle and medial tibial plateau; (**b**) Postoperative alignment of the lower limb was unsatisfactory (WBL ratio, 47.1%); (**c**) Second-look arthroscopy shows sufficient restoration of the articular cartilage at postoperative 23 months. The postoperative Knee Society knee and function scores were 55 and 60 points, respectively, and the postoperative Western Ontario and McMaster Universities total score was 29 points, all of which were unsatisfactory

Several articles have investigated the effect of simple HTO without any cartilage repair procedures on clinical outcomes [30, 31]. Spahn et al. [30] found that a patient history of more than 24 months, a poor preoperative clinical score, obesity, and smoking were associated with a worse outcome after HTO alone at midterm follow-up. In another study, age > 56 years and postoperative knee flexion less than 120° were significantly related to a poor outcome after simple HTO (*p* = 0.008 and *p* < 0.001, respectively), whereas the Ahlbäck grade of medial compartment arthritis and excellent preoperative KS scores were significantly related to a better outcome after simple HTO (*p* < 0.001 and *p* < 0.001, respectively) [31]. However, it has remained unclear which preoperative and postoperative factors affect clinical outcomes after HTO combined with a cartilage procedure. In our study, although no statistical results were obtained, a number of patients, whose cartilage regeneration did not agree with the improvement in clinical outcome after OW HTO with microfrature, have been identified (Figs. 2 and 3).

In terms of the MOCART score after HTO, Verdonk et al. [32] reported that complete integration to the border zone was found in 25% of patients at postoperative 2 years. However, Kim et al. [17] reported complete integration in 1 patient out of 14 patients with microfracture alone at 1-year follow-up. Our study also showed very low incidence of integration, which was consistent with the findings of Kim et al. (Table 3). Kim et al. [17] reported no hypertrophy of the cartilage, and subchondral bone marrow edema was found in 78.6% of patients with OW HTO plus microfracture. Conversely, in our study, hypertrophy of the cartilage was observed in 28.1% of patients, whereas subchondral bone marrow edema was found in only 29.8% of patients with OW HTO plus microfracture. These results could be attributed to the fact that the follow-up period of our study was 2 years, which was markedly longer than the 1-year follow-up of the study by Kim et al.

There are several limitations in this study. First, the follow-up period was only 2 years after surgery. A long-term investigation is necessary to determine whether the changes in articular cartilage affect survival after OW HTO. Second, a comparison has not been performed between the under-correction and over-correction groups. Degree of the correction could affect the biological regeneration of the articular cartilage after OW HTO due to the different loading on the medial compartment. However, there were few outliers under 55% or over 70% of the WBL ratio, so we assumed that degree of the correction would have little effect on the results. Third, the postoperative rehabilitation protocol differed between the patients with and without a microfracture. The differences in the timing of postoperative weight-bearing could affect the regeneration of cartilage.

## Conclusion

Microfracture of the MFC during OWHTO only helped the filling of the degenerative cartilage defect and the integration of the cartilage with adjacent cartilage. However, the clinical and radiologic outcome could not be improved by mircrofracture in the OWHTO.

## Abbreviations

HKA: hip-knee-ankle
HTO: high tibial osteotomy
ICRS: International Cartilage Repair Society
JLCA: joint line convergence angle
KS: Knee Society
LFC: lateral femoral condyle
LTP: lateral tibial plateau
MFC: medial femoral condyle
MOCART: magnetic resonance observation of cartilage repair tissue
MRI: magnetic resonance imaging
MTP: medial tibial plateau
OW: open wedge
SD: standard deviation
WBL: weight-bearing line
WOMAC: Western Ontario and McMaster University
